# Mitigating environmental public health risks via artificial intelligence: mechanisms and boundary conditions

**DOI:** 10.3389/fpubh.2026.1889267

**Published:** 2026-07-22

**Authors:** Yushan Qiu, Siyuan Huang, Wenjing Deng, Joston Gary

**Affiliations:** 1 School of Economics and Management, Jiangxi Normal University, Nanchang, China; 2 School of Journalism and Communication, Guangxi University, Nanning, China; 3 Department of Management and Engineering, Linköping University, Linköping, Sweden; 4 Department of Economics, Management, Industrial Engineering and Tourism, University of Aveiro, Aveiro, Portugal; 5 School of Economics, Management and Political Science, University of Minho, Braga, Portugal

**Keywords:** artificial intelligence, environmental investment, environmental public health risk, environmental regulation, pollution mitigation, technological expenditure

## Abstract

**Background:**

Environmental pollution threatens population health through multiple and overlapping pathways, including gaseous emissions, wastewater discharge, and industrial solid waste. Artificial intelligence (AI) can improve environmental monitoring, energy management, and production optimization, but its broader relationship with multidimensional environmental public health risks remains insufficiently understood. This study examines whether and under what conditions artificial intelligence is associated with lower pollution-related environmental public health risks.

**Methods:**

Provincial panel data covering 30 regions in China from 2014 to 2023 were analyzed. A multidimensional environmental public health risk index was constructed from carbon dioxide emissions, sulfur dioxide emissions, nitrogen oxide emissions, industrial wastewater discharge, and industrial solid waste. Two-way fixed-effects models were combined with mediation analysis, heterogeneity and marginal-effect analysis, alternative measurement, additional control and winsorization tests, dynamic panel estimation, and a panel threshold model.

**Results:**

Higher levels of artificial intelligence were associated with lower environmental public health risks in the principal fixed-effects models, and the negative relationship remained consistent across alternative measurement, additional digital-infrastructure controls, winsorization, and concurrent policy specifications. Technological expenditure emerged as an implementation pathway through which digital capability can be translated into monitoring systems, cleaner equipment, and environmental management infrastructure, while green patents reflected a longer-horizon innovation process. Environmental investment strengthened the negative association by providing the financial and physical capacity required for artificial intelligence deployment. Electricity consumption identified greater potential for energy and production optimization, although the contribution of artificial intelligence varied across energy-use conditions. Artificial intelligence remained negatively associated with environmental public health risks across environmental regulation regimes, while its marginal contribution changed non-linearly with regulatory intensity.

**Conclusion:**

Artificial intelligence functions as a conditional environmental capability rather than an automatic technological solution. Its public health value is more likely to emerge when digital development is supported by technological expenditure, environmental investment, operational implementation, and coordinated regulatory design. These findings provide a multidimensional framework for understanding how artificial intelligence can contribute to pollution-related environmental public health risk mitigation.

## 1 Introduction

Environmental pollution remains a major source of preventable public health risk. Industrial production releases pollutants through the atmosphere, water systems, and solid waste streams, creating several routes through which environmental conditions may affect population health. Premature deaths associated with modern forms of pollution have increased substantially in recent decades, while air pollution and unsafe water continue to impose considerable health burdens across countries (1–3). These risks are especially difficult to assess in industrializing regions because different pollutants often occur simultaneously. An evaluation based on a single emission category may therefore provide an incomplete account of the environmental conditions associated with public health risk.

Artificial intelligence (AI) offers several technical functions that may help reduce pollution-related risks. Intelligent systems can improve pollutant monitoring, identify abnormal emission patterns, predict changes in air quality, and support the allocation of energy and environmental resources (4–7). Artificial intelligence may also support cleaner production by improving energy management, facilitating environmental data integration, and accelerating green technological development (5–7). These capabilities suggest that artificial intelligence may be associated with lower environmental public health risks. Existing evidence, however, has largely examined individual pollutants, carbon emissions, or specific technical applications. It remains uncertain whether the development of artificial intelligence corresponds to a broader reduction in multiple pollution-related risks at the regional level.

China provides a suitable setting for examining this question. Its provincial regions differ considerably in industrial structure, technological capacity, energy use, environmental investment, and regulatory intensity. These differences create substantial regional variation in both artificial intelligence development and pollution-related environmental conditions. China also has an important position in global hazardous emissions and related health burdens, which gives the study of its regional exposure control policies wider public health relevance (8). Environmental health policies in China have traditionally focused on air and water pollution, while public concern is also highest for these two forms of environmental exposure (9, 10). At the same time, the effectiveness of existing pollution control measures varies across regions and may depend on local environmental and institutional conditions (11, 12). This regional variation makes China an appropriate context for examining whether artificial intelligence is associated with lower environmental public health risks and whether this relationship depends on local resources and regulatory conditions.

**Figure 1:**
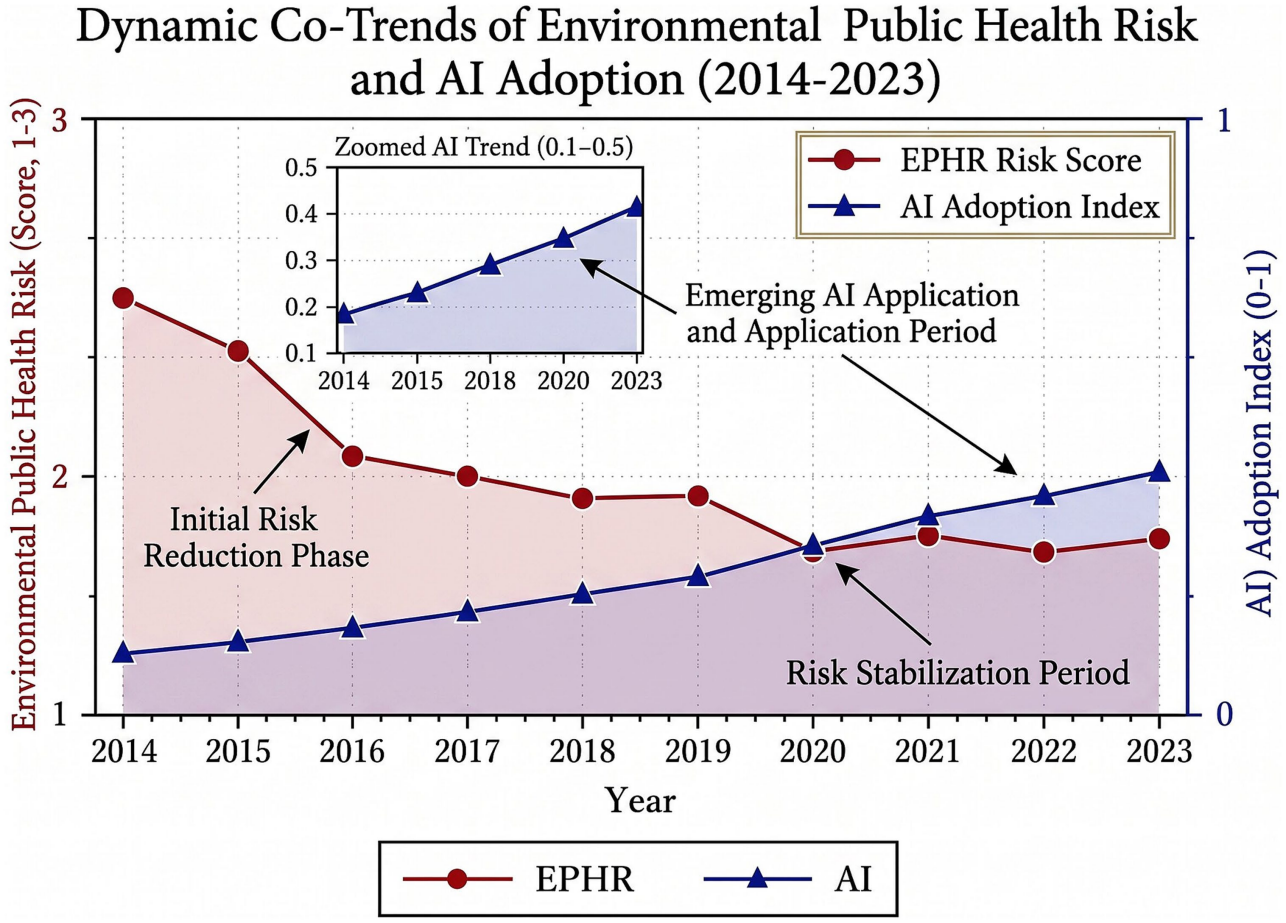
National dynamic trends in artificial intelligence and environmental public health risks in China, 2014–2023.

Figure 1 presents the national dynamic trends in artificial intelligence and environmental public health risks from 2014 to 2023. Artificial intelligence increased during this period, while the environmental public health risk index generally declined. These parallel trends provide descriptive motivation for the study, although they do not establish an independent relationship between the two variables. A systematic analysis is needed to distinguish the association of artificial intelligence from persistent provincial characteristics, national time trends, economic conditions, and environmental policy differences.

The existing literature leaves four related questions unresolved. First, research on artificial intelligence and environmental performance has not reached a consistent conclusion. Artificial intelligence may improve monitoring, production efficiency, and energy allocation, but the operation of digital infrastructure may also increase energy demand and environmental pressure (4–7, 13). The net regional relationship therefore remains open to empirical examination. Second, much of the existing literature evaluates environmental performance through a single pollutant or emission category. Such measures do not fully represent the combined environmental conditions created by gaseous emissions, wastewater discharge, and industrial solid waste. Third, the processes linking artificial intelligence to lower environmental risk remain insufficiently examined. Technological expenditure may convert digital capacity into monitoring systems, cleaner equipment, and environmental infrastructure, while green patents may represent innovation outputs that support cleaner production. Fourth, the effect of artificial intelligence may depend on regional conditions. Environmental investment can support the implementation of intelligent environmental systems, electricity consumption reflects the scale and energy intensity of regional production, and environmental regulation may alter the benefits and constraints associated with artificial intelligence.

This study addresses these questions using panel data from 30 provincial regions in China between 2014 and 2023. Environmental public health risk is represented by a composite index constructed from industrial sulfur dioxide emissions, carbon dioxide emissions, industrial wastewater discharge, nitrogen oxide emissions, and industrial solid waste. These indicators cover atmospheric, aquatic, and solid waste conditions associated with potential public health risks. Because the indicators are correlated and measured on different scales, a principal component-based procedure is used to reduce dimensionality and derive data-based weights without imposing a subjective weighting scheme (14). The index represents regional pollution-related environmental conditions associated with potential health risks; it does not directly measure individual exposure, disease incidence, or mortality. The study then examines the relationship between artificial intelligence and this index, assesses technological expenditure and green patents as proposed transmission mechanisms, and evaluates whether the relationship varies with environmental investment, electricity consumption, and environmental regulation.

This study makes three contributions. First, it connects artificial intelligence development with a multidimensional measure of pollution-related environmental public health risk at the provincial level. This approach extends research that has concentrated mainly on carbon emissions or individual pollutants. Second, it develops an index that combines atmospheric emissions, wastewater discharge, and industrial solid waste, providing a broader regional measure of environmental conditions associated with potential public health risk. Third, it examines both proposed mechanisms and regional conditions. The analysis distinguishes the roles of technological expenditure and green patents and evaluates how environmental investment, electricity consumption, and regulatory intensity shape the association between artificial intelligence and environmental public health risks. These contributions provide a clearer account of when and through which processes artificial intelligence may support environmental risk reduction.

## 2 Theoretical analysis and hypothesis development

Environmental public health risks arise from the combined effects of atmospheric emissions, wastewater discharge, and industrial solid waste. These environmental pressures can occur simultaneously and affect population health through different exposure routes. A single pollutant, therefore, cannot fully represent the regional burden associated with environmental degradation (15, 16). Composite measurements can better reflect the joint influence of multiple environmental hazards, especially when gaseous emissions, water pollution, and solid waste coexist within the same region (17).

Artificial intelligence differs from conventional computing systems through its capacity to process large volumes of data, identify complex patterns, update predictions, and support decisions under uncertainty (18). These functions are directly relevant to environmental monitoring, production management, energy allocation, and pollution control. The following sections explain the expected relationship between artificial intelligence and environmental public health risks, the mediating roles of technological expenditure and green patents, and the regional conditions that may shape this relationship.

### 2.1 Artificial intelligence and environmental public health risks

Artificial intelligence can reduce environmental public health risks through improved information processing and operational optimization. Environmental management depends on the timely identification of pollution sources, accurate forecasting of environmental changes, and efficient allocation of technical and financial resources. Conventional monitoring systems may be limited by delayed reporting, fragmented databases, and weak predictive capacity. Artificial intelligence can integrate data from industrial sensors, geographic information systems, environmental monitoring networks, and administrative records, allowing authorities and firms to detect abnormal emissions and respond more quickly (19).

This information processing capacity is relevant to both water and air pollution. Artificial intelligence-based systems can identify contamination patterns, locate potential pollution sources, and support targeted treatment of polluted water bodies (20). Machine learning models can also forecast pollutant concentrations, identify high-risk periods, and improve early warning systems for environmental conditions associated with respiratory and cardiovascular harm (21). Faster detection and prediction can reduce the delay between the generation of hazardous emissions and the implementation of control measures.

Artificial intelligence may also reduce environmental pressure through production and energy optimization. Intelligent systems can improve transportation routes, equipment operation, production scheduling, and energy allocation, thereby lowering unnecessary resource use and emissions (22). In the energy sector, artificial intelligence can support load forecasting, power system coordination, and the integration of cleaner energy sources (23). At the firm level, these functions can identify inefficient processes, predict equipment failure, and reduce operational waste.

The environmental effect of artificial intelligence depends on whether these efficiency gains exceed the energy and infrastructure costs associated with digital systems. Artificial intelligence requires computing power, data centers, network infrastructure, and technical personnel. Its net environmental effect is therefore not automatic. When artificial intelligence is applied to environmental monitoring, energy management, and production control, it can improve the identification of pollution sources, reduce operational waste, and support lower-emission decisions (19–24). This reasoning leads to the following hypothesis:

*Hypothesis 1*: Artificial intelligence is negatively associated with environmental public health risks.

### 2.2 Mediating roles of technological expenditure and green patents

Artificial intelligence may affect environmental public health risks through changes in technological inputs and innovation outputs. Technological expenditure reflects the financial resources allocated to technological development and application. Green patents represent formalized outputs of environmentally oriented innovation. These two variables capture different stages of the technological development process. Technological expenditure supports implementation, while green patents represent technical solutions that may contribute to cleaner production and pollution control.

#### 2.2.1 Mediating role of technological expenditure

The development and application of artificial intelligence require sustained expenditure on research, computing infrastructure, software, technical personnel, data systems, and industrial equipment. Stronger artificial intelligence capacity may increase regional demand for technological expenditure because intelligent systems require continuous investment for development, deployment, and maintenance (25, 26). Artificial intelligence can also improve the allocation of technological funds by identifying pollution sources, evaluating technical alternatives, and directing resources toward projects with greater environmental value.

Technological expenditure can reduce environmental public health risks when it finances environmental monitoring systems, cleaner equipment, pollution treatment technologies, and energy management infrastructure. Investment in sensors and data platforms can improve the detection of hazardous emissions, while investment in automated equipment can improve the control of industrial processes (27, 28). Continued expenditure is also necessary for software updates, equipment replacement, data maintenance, and technical training.

The environmental value of technological expenditure depends on how financial resources are allocated. General technological spending does not necessarily produce pollution reduction. Artificial intelligence can improve the environmental effectiveness of this spending by supporting project selection, forecasting expected outcomes, and identifying sectors with high pollution control needs. Technological expenditure can then be converted into monitoring networks, cleaner production equipment, and energy management systems that reduce hazardous emissions (29–32).

The proposed mechanism therefore contains two relationships. Artificial intelligence is expected to increase or improve the allocation of technological expenditure. Technological expenditure is then expected to support environmental infrastructure and cleaner production systems that reduce environmental public health risks. Accordingly:

*Hypothesis 2a*: Technological expenditure mediates the negative relationship between artificial intelligence and environmental public health risks.

#### 2.2.2 Mediating role of green patents

Artificial intelligence may also affect environmental public health risks through green innovation. Green technological development involves high research costs, long testing periods, and uncertainty concerning technical outcomes. Artificial intelligence can support simulation, design optimization, data analysis, and the identification of promising technical solutions, thereby reducing trial costs and shortening development cycles (33).

Green patents represent codified technological outputs related to pollution control, energy efficiency, cleaner production, and resource conservation. Patent activity does not guarantee immediate environmental improvement, since technical knowledge must be adopted and applied before it changes production outcomes. Once green patented technologies are used by firms or public agencies, they can support cleaner industrial processes, improved pollution treatment methods, and more efficient transportation and energy systems (34, 35).

Artificial intelligence can enhance the speed and precision of green research and development. Predictive models can help identify effective materials, production methods, and emission control solutions. Intelligent automation can also improve coordination across design, testing, and industrial application (36–38). When these technologies are adopted in production, they may reduce resource use and hazardous emissions, thereby lowering pollution-related environmental public health risks (39).

The proposed mechanism therefore assumes that artificial intelligence supports the development of green patents and that the application of green patented technologies contributes to lower environmental risk. Based on this reasoning:

*Hypothesis 2b*: Green patents mediate the negative relationship between artificial intelligence and environmental public health risks.

**Figure 2:**
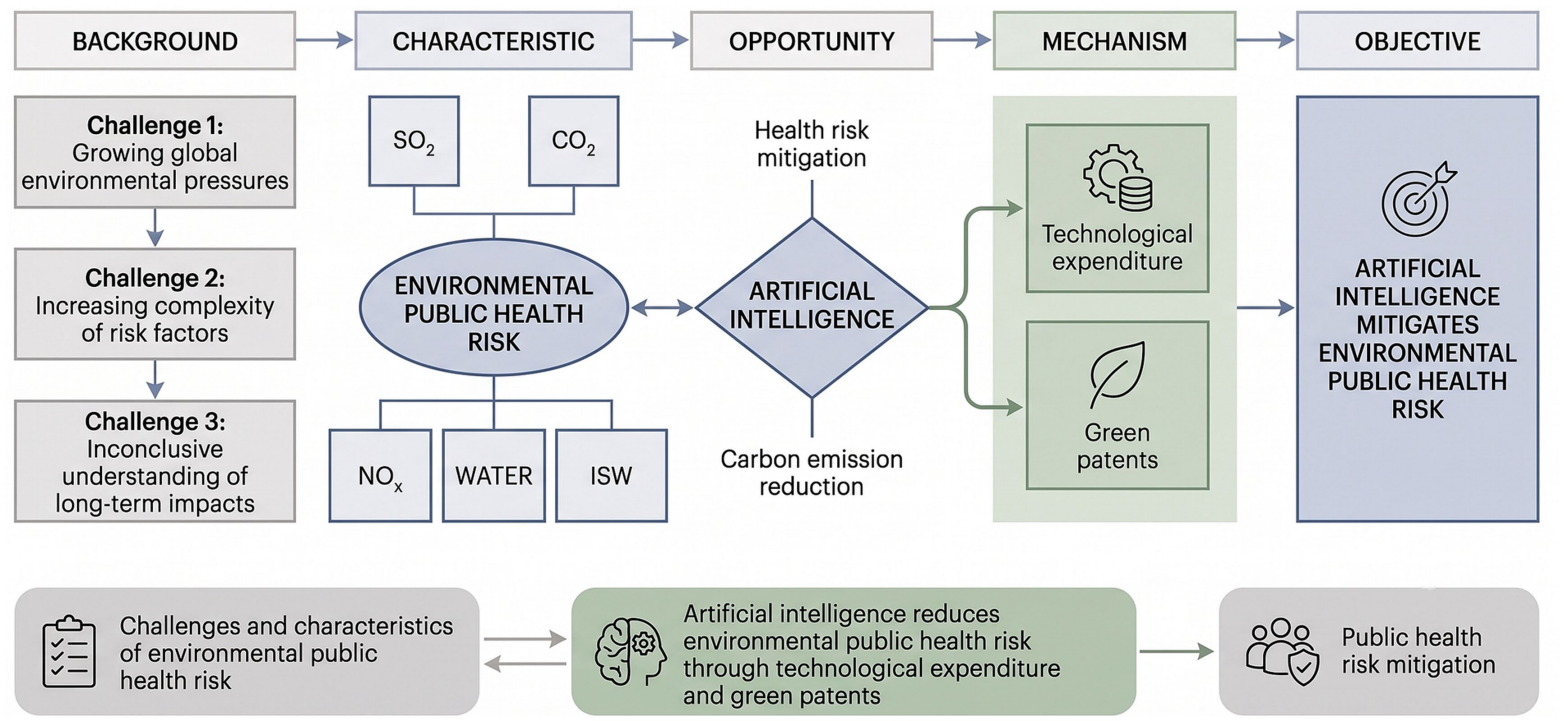
Proposed mediation pathways linking artificial intelligence with environmental public health risks.

Figure 2 presents the proposed transmission paths through technological expenditure and green patents.

### 2.3 Heterogeneity across environmental investment and electricity consumption

The relationship between artificial intelligence and environmental public health risks may vary across regional conditions. Artificial intelligence applications depend on financial resources, environmental infrastructure, energy systems, and the scale of regional production. Environmental investment and electricity consumption capture two aspects of this regional context.

Environmental investment reflects the financial resources allocated to pollution control, environmental infrastructure, ecological restoration, and related public programs. Artificial intelligence-based environmental systems require monitoring equipment, data platforms, computing capacity, and technical maintenance. Regions with higher environmental investment are more capable of financing these requirements and integrating intelligent technologies into environmental management systems (40, 41).

Environmental investment can also reduce the gap between technical capacity and practical implementation. Without sufficient funding, artificial intelligence may remain limited to planning, testing, or isolated applications. Higher environmental investment allows local governments and firms to convert digital capacity into monitoring networks, treatment equipment, and cleaner production systems (42, 43). In regions with limited environmental investment, weak infrastructure and insufficient implementation capacity may restrict the environmental benefits associated with artificial intelligence (44).

This reasoning suggests that artificial intelligence should be more strongly associated with lower environmental public health risks where environmental investment is higher. Accordingly:

*Hypothesis 3a*: The negative association between artificial intelligence and environmental public health risks is stronger in regions with higher environmental investment.

Electricity consumption reflects the scale and energy intensity of regional economic activity. High electricity consumption is often associated with larger industrial systems, greater energy dependence, and more complex production processes. These conditions create broader opportunities for artificial intelligence to improve load forecasting, equipment scheduling, predictive maintenance, and production coordination (45, 46).

The potential environmental gain from optimization may be greater where baseline energy use and operational inefficiency are high. Artificial intelligence can identify unnecessary electricity use, improve dynamic energy allocation, and support the integration of cleaner energy sources within complex production and power systems (32, 47). High-energy demand regions provide greater scope for operational improvement because inefficient load allocation, equipment use, and production scheduling create larger potential gains from intelligent optimization (48).

In regions with lower electricity consumption, the scale of energy-related optimization may be smaller, and the environmental benefits of artificial intelligence less pronounced. This leads to the following hypothesis:

*Hypothesis 3b*: The negative association between artificial intelligence and environmental public health risks is stronger in regions with higher electricity consumption.

### 2.4 Non-linear role of environmental regulation

Environmental regulation may change how artificial intelligence is applied to pollution control. Regulatory pressure affects firms’ incentives to monitor emissions, invest in cleaner equipment, disclose environmental information, and comply with pollution standards. When environmental regulation is weak, firms may have limited incentive to direct artificial intelligence toward environmental applications. Intelligent systems may instead be used mainly to improve production efficiency or reduce operating costs.

As environmental regulation strengthens, firms and local governments face greater pressure to monitor pollution and improve environmental performance. This pressure can increase demand for artificial intelligence-based monitoring, forecasting, production control, and compliance systems. Environmental regulation can therefore influence whether artificial intelligence is used mainly for production growth or for pollution reduction (49, 50).

The effect of environmental regulation may not increase at a constant rate. At lower levels, incremental regulatory changes may produce limited environmental responses because enforcement capacity, technical infrastructure, and organizational compliance remain insufficient. Once regulation reaches a level that materially affects firm behavior, artificial intelligence may become more closely integrated into pollution control decisions. At higher levels, additional regulation may generate compliance costs, reduce available investment resources, or produce diminishing marginal gains. These competing effects imply that the association between artificial intelligence and environmental public health risks may vary across regulatory levels (51, 52).

The threshold argument therefore concerns the regulatory conditions under which artificial intelligence is applied. Environmental regulation can change firms’ incentives, implementation costs, and demand for digital environmental systems. Based on this reasoning:

*Hypothesis 4*: The association between artificial intelligence and environmental public health risks varies non-linearly across levels of environmental regulation.

**Figure 3:**
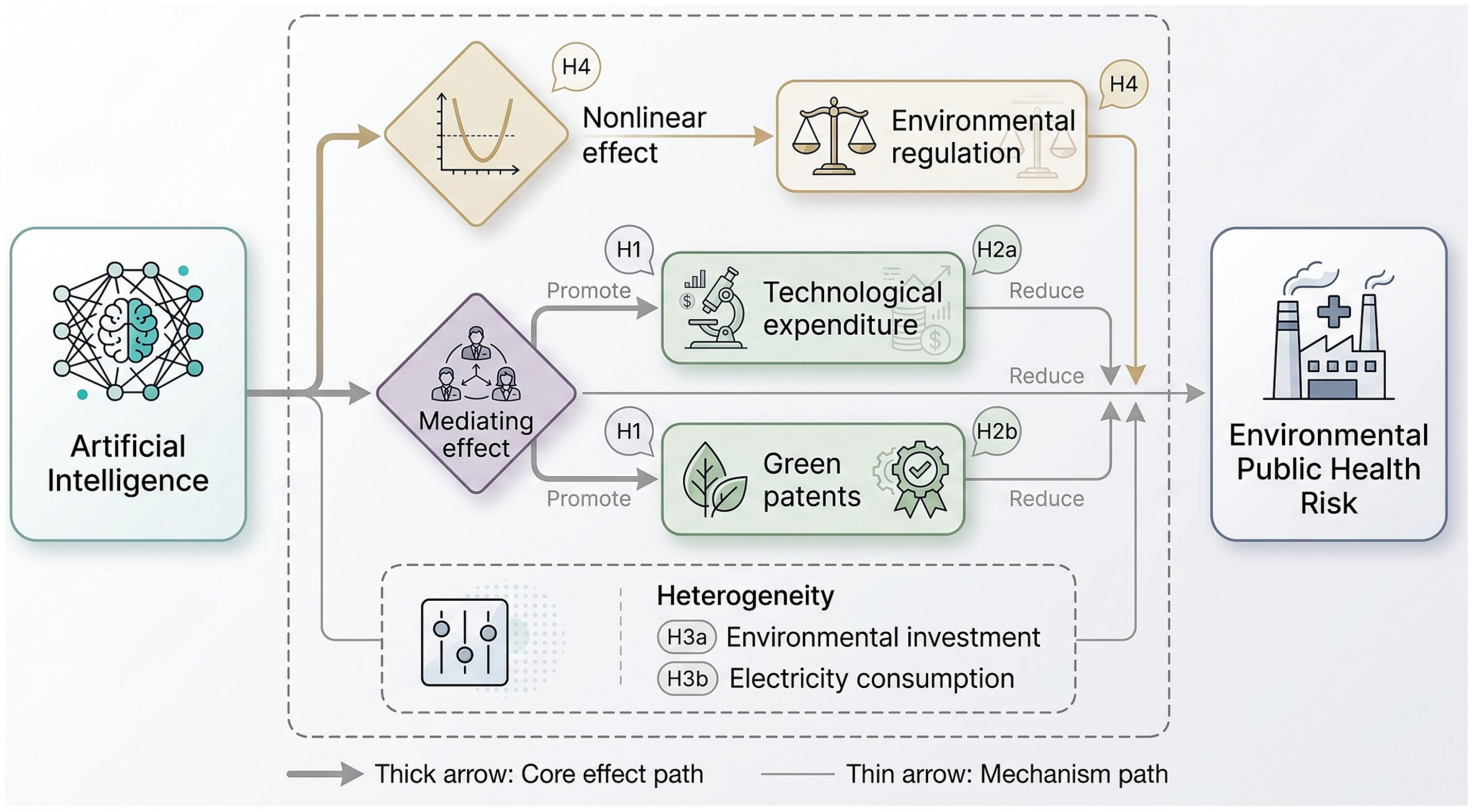
Conceptual model of the association between artificial intelligence and environmental public health risks.

Figure 3 presents the full conceptual model, including the direct relationship, the proposed mediating mechanisms, the regional heterogeneity conditions, and the non-linear role of environmental regulation.

## 3 Research model and data

### 3.1 Model specification

This study uses a two-way fixed-effects panel model to examine the association between artificial intelligence and environmental public health risks. The baseline model is specified as follows:

$$\mathrm{EPH}R_{\mathrm{it}}=\alpha_{0}+\alpha_{1}AI_{\mathrm{it}}+\theta^{\prime }C_{\mathrm{it}}+\mu_{i}+\lambda_{t}+\varepsilon_{\mathrm{it}}$$ (1)

In Equation 1, (i) indexes provincial regions and (t) indexes years. (EPHR_it_) denotes the environmental public health risk index, while (AI_it_) denotes the artificial intelligence index. (C_it_) represents the vector of control variables, including environmental regulation, industrial agglomeration, industrialization level, and human capital. Provincial fixed effects are represented by (μ_i_), year fixed effects are represented by (λ_t_), and (ε_t_) is the error term.

Provincial fixed effects account for time-invariant differences across regions, including persistent geographic, industrial, and institutional characteristics. Year fixed effects account for national economic changes, policy developments, and other common shocks affecting all provincial regions during the same year. This specification reduces bias associated with these two sources of unobserved heterogeneity and is suitable for the balanced provincial panel used in this study (53).

Several diagnostic and supplementary analyses evaluate the empirical specification. Panel unit root tests examine the time-series properties of the variables. A residual cross-sectional dependence test evaluates whether common regional correlations remain after estimation. The Kao test is reported as supplementary evidence concerning the long-run relationship among the variables and is not treated as a prerequisite for the fixed-effects estimation (54). The stability of the baseline findings is further evaluated through alternative measures, additional control variables, a dynamic panel model, and controls for concurrent national policies.

Artificial intelligence may also be associated with environmental public health risks through technological expenditure and green patents. The mediation equations are specified as follows:

$$M_{\mathrm{it}}=\beta_{0}+\beta_{1}AI_{\mathrm{it}}+\theta_{M}^{\prime }C_{\mathrm{it}}+\mu_{i}+\lambda_{t}+\nu_{\mathrm{it}}$$ (2)

$$\mathrm{EPH}R_{\mathrm{it}}=\gamma_{0}+\gamma_{1}AI_{\mathrm{it}}+\gamma_{2}M_{\mathrm{it}}+\theta_{Y}^{\prime }C_{\mathrm{it}}+\mu_{i}+\lambda_{t}+\xi_{\mathrm{it}}$$ (3)

In Equations 2 and 3, (M_it_) represents the mediating variable, specified separately as technological expenditure or green patents. Equation 2 estimates the association between artificial intelligence and the mediator. Equation 3 estimates the associations of artificial intelligence and the mediator with environmental public health risks. The indirect effect is calculated as follows:

$$\mathrm{IE}=\beta_{1}\gamma_{2}$$

The statistical stability of the indirect effects is evaluated using 5,000 province-clustered Bootstrap resamples. Each Bootstrap replication re-estimates Equations 2 and 3 while retaining provincial fixed effects, year fixed effects, and the same control variables used in the baseline model. Province-level clustering accounts for dependence among annual observations from the same region. Percentile and bias-corrected and accelerated 95% confidence intervals are reported.

For comparison, pooled mediation models without provincial and year fixed effects are presented in Appendix A. The pooled models combine persistent differences across provincial regions with temporal variation within provincial regions. They are reported as a comparison and are not used as the primary basis for evaluating Hypotheses 2a and 2b. The heterogeneous roles of environmental investment and electricity consumption are examined using interaction terms:

$$\begin{array}{c} \mathrm{EPH}R_{\mathrm{it}}=\delta_{0}+\delta_{1}AI_{\mathrm{it}}+\delta_{2}Z_{\mathrm{it}}+\delta_{3}(AI_{\mathrm{it}}\times Z_{\mathrm{it}})+\theta_{Z}^{\prime }C_{\mathrm{it}}\\ +\mu_{i}+\lambda_{t}+\omega_{\mathrm{it}} \end{array}$$ (4)

In Equation 4, (Z_it_) represents the regional condition used in the heterogeneity analysis. It is specified separately as environmental investment (EI_it_) or electricity consumption (EC_it_). The coefficient (δ_3_) captures the interaction between artificial intelligence and the corresponding regional condition. A negative value of (δ_3_) indicates that the negative association between artificial intelligence and environmental public health risks becomes stronger as environmental investment or electricity consumption increases. Environmental regulation may produce a non-linear change in the association between artificial intelligence and environmental public health risks. The single-threshold model is specified as follows:

$$\begin{array}{c} \mathrm{EPH}R_{\mathrm{it}}=\phi_{0}+\phi_{1}AI_{\mathrm{it}}I(ER_{\mathrm{it}}\leq \eta )+\phi_{2}AI_{\mathrm{it}}I(ER_{\mathrm{it}}>\eta )\\ +\theta_{T}^{\prime }C_{\mathrm{it}}+\mu_{i}+\lambda_{t}+\varepsilon_{\mathrm{it}} \end{array}$$ (5)

In Equation 5, (ER_it_) represents environmental regulation and (θ) denotes the estimated threshold value. (I(\cdot)) is an indicator function that equals one when the stated condition is satisfied and zero otherwise. The coefficients (Φ_1_) and (Φ_2_) represent the association between artificial intelligence and environmental public health risks below and above the estimated environmental regulation threshold, respectively. Single-, double-, and triple-threshold specifications are tested sequentially. Only threshold structures supported by Bootstrap significance tests are used for interpretation.

### 3.2 Variable definitions

This study utilizes a balanced panel dataset comprising 30 provincial regions in China, spanning the period from 2014 to 2023. The selection of this specific timeframe is carefully considered based on concurrent technological and regulatory milestones. The year 2014 marks a critical turning point in the national strategic elevation of big data and the large-scale rollout of digital infrastructure, which provided the essential data accumulation required for the subsequent growth of artificial intelligence. Simultaneously, 2014 aligns with the comprehensive implementation of stricter environmental monitoring networks across Chinese provinces, ensuring the availability and consistency of high-resolution environmental data. Ending the sample period in 2023 ensures the incorporation of recent macroeconomic statistics, providing a reasonably contemporary context to observe the ongoing environmental effects of intelligent technologies. Based on this sample pool, the specific variables are constructed as follows.

#### 3.2.1 Explained variable

The explained variable is environmental public health risk. Industrial sulfur dioxide emissions, industrial wastewater discharge, industrial smoke and dust, and other industrial waste indicators have been used to represent pollution-related environmental conditions that may affect population health (55). A single pollutant, however, cannot fully reflect the combined environmental pressure created by atmospheric emissions, aquatic pollution, and solid waste. This study therefore constructs a composite environmental public health risk index from five indicators: industrial sulfur dioxide emissions, carbon dioxide emissions, industrial wastewater discharge, industrial nitrogen oxide emissions, and industrial solid waste.

Sulfur dioxide and nitrogen oxides represent hazardous atmospheric emissions with direct relevance to respiratory health. Carbon dioxide represents climate-related environmental pressure associated with fossil energy use and industrial activity. Industrial wastewater captures the aquatic dimension of regional pollution, while industrial solid waste captures environmental pressure arising from the production, storage, and disposal of industrial residues. The joint use of these indicators reflects that environmental health risks may arise through several exposure routes and may accumulate within the same region (56).

Industrial smoke and dust are not included because changes in official statistical definitions and reporting standards reduce their comparability across the study period. Provincial carbon dioxide emissions are obtained from the Emissions Database for Global Atmospheric Research because a complete and consistent provincial series is not available from the official Chinese statistical yearbooks. The data processing procedure follows the approach applied by Lekaki et al. (57). Industrial sulfur dioxide emissions, industrial wastewater discharge, industrial nitrogen oxide emissions, and industrial solid waste are obtained from Chinese environmental and regional statistical sources.

A principal component factor method is applied to combine the five indicators. This method reduces the dimensionality of the correlated variables and produces data-based weights without imposing a subjective weighting structure. The five indicators are standardized before factor extraction. Two factors with eigenvalues greater than one are retained. Their eigenvalues are 3.068 and 1.263, and they jointly explain 86.63% of the total variance. The Kaiser–Meyer–Olkin value is 0.612. Regression factor scores are generated for the two retained factors. Their weights are calculated from their respective shares of the retained eigenvalues:

$$w_{1}=\frac{3.068}{3.068+1.263}=0.708329,$$

$$w_{2}=\frac{1.263}{3.068+1.263}=0.291671.$$

The environmental public health risk index is calculated using Equation 6 as follows:

$$\mathrm{EPH}R_{\mathrm{it}}=2+0.708329z_{1,\mathrm{it}}+0.291671z_{2,\mathrm{it}}$$ (6)

where (z_1,it_) and (z_2,it_) denote the regression factor scores for provincial region (i) in year (t). A constant of two is added to ensure positive index values. This translation changes the location and intercept of the index but does not change its correlations or slope coefficients.

The index represents regional pollution-related environmental conditions associated with potential public health risks. It does not directly measure individual exposure, disease incidence, hospitalization, or mortality. Table 1 presents the environmental public health risk indicator system, while Table 2 reports the principal component factor extraction results.

**Table 1:** Environmental public health risks indicator system.

Explained variable	Primary indicator	Symbol
Environmental Public Health Risk (EPHR)	Industrial sulfur dioxide emissions	$SO_{2}$
	Carbon dioxide releases	$CO_{2}$
	Industrial wastewater discharges	WATER
	Industrial atmospheric nitrogen oxide content	$NO_{x}$
	Industrial solid waste	ISW

**Table 2:** Principal component factor extraction results.

Factor	Eigenvalue	Proportion	Cumulative	$SO_{2}$	$CO_{2}$	Water	$NO_{x}$	ISW
Factor1	3.068	0.614	0.614	0.897	0.662	0.784	0.967	0.526
Factor2	1.263	0.253	0.866	−0.322	0.560	−0.513	−0.081	0.759
Factor3	0.46	0.0920	0.958					
Factor4	0.162	0.032	0.991					
Factor5	0.047	0.009	1.000					
Uniqueness	-	-	-	0.094	0.248	0.121	0.058	0.148
KMO	0.612							

#### 3.2.2 Explanatory variable

The primary explanatory variable is artificial intelligence. Three general approaches can be used to measure artificial intelligence development. Bibliometric measures use artificial intelligence-related keywords, publications, or textual frequency to represent research activity and public attention (58, 59). Physical deployment measures use indicators such as industrial robot installation or robot density to represent the application of intelligent production technologies (38, 60). Robot-based measures are useful for assessing manufacturing automation and its environmental effects, although they do not capture artificial intelligence development in research, finance, software, and other economic activities (61).

A multidimensional index provides a broader measure of provincial artificial intelligence development because it combines research inputs, financial resources, and applied outputs (62). Following this logic, the artificial intelligence index is constructed from three dimensions: research capability, financial support, and research development. These dimensions represent successive parts of the regional technological development process and are consistent with the structure used to evaluate regional differences in artificial intelligence development (63).

Research capability is measured using the number of research and development projects in universities, the number of research and development institutions in major industrial enterprises, and the number of employees in information transmission and software-related sectors. These indicators represent regional research infrastructure, technical labor, and digital sector capacity.

Financial support is measured using internal research and development expenditure, funding expenditure by research institutions, and internal research and development expenditure in high-technology industries. These indicators capture the financial resources available for computing facilities, technical research, product development, and the application of intelligent technologies.

Research development is measured using transaction turnover in technology markets, the number of patent applications by high-technology enterprises, and the number of scientific papers published by universities. These indicators represent the conversion of research and financial inputs into transferable knowledge, technological applications, and formal innovation outputs.

All indicators are treated as positive and normalized before the entropy weights are calculated. The entropy procedure assigns greater weights to indicators that provide more information variation across provincial regions and years. The weighted indicators are then aggregated into the artificial intelligence index. Higher index values indicate higher levels of regional artificial intelligence development. Table 3 reports the three dimensions and their constituent indicators.

**Table 3:** Artificial intelligence indicator system.

Explanatory variables	Primary indicators	Secondary indicators
Artificial Intelligence (AI)	Research capability	Number of research and development projects in universities
		Number of research and development institutions in major industrial enterprises
		Number of employees in information transmission and software sectors
	Financial support	Internal expenditure on research and development
		Funding expenditure of research institutions
		Internal research and development expenditure in high technology industries
	Research development	Transaction turnover in technology markets
		Number of patent applications by high technology enterprises
		Scientific papers published by universities

#### 3.2.3 Mediating variables

The mechanism analysis examines technological expenditure and green patents as mediating variables. Technological expenditure is measured by the aggregate regional expenditure on science and technology. It represents the financial resources allocated to technological development, technical services, equipment, and implementation. Technological expenditure can support regional innovation capacity, technological capital accumulation, cleaner production systems, and industrial environmental improvement (64). In the present analysis, it is treated as an aggregate regional expenditure variable. The artificial intelligence index, in comparison, is constructed from several specific indicators across research capability, financial support, and research development.

Green patents are measured by the number of green patent applications. Green patent applications represent formalized innovation outputs related to pollution control, energy efficiency, cleaner production, and resource conservation. They provide a measurable indication of regional activity in environmentally oriented technological development (65).

#### 3.2.4 Control variables

The baseline model includes environmental regulation, industrial agglomeration, industrialization level, and human capital. These variables capture regional regulatory, economic, industrial, and educational conditions that may be associated with environmental public health risks (66, 67).

Environmental regulation is measured as completed investment in industrial pollution control divided by industrial value added. The ratio represents the relative intensity of regional resources allocated to industrial pollution treatment. A higher value indicates that a larger share of industrial economic activity is accompanied by pollution control investment.

Industrial agglomeration is proxied by employment density and is calculated as total employment divided by administrative land area. The measure captures the spatial concentration of economic activity. Higher concentration may increase local pollution pressure through production clustering, while shared infrastructure and technical services may also improve the efficiency of pollution treatment.

Industrialization level is measured as industrial value added divided by regional gross domestic product. This variable controls for differences in the economic importance of industrial production across provincial regions. Regions with a larger industrial sector may face higher environmental pressure because industrial activity remains a major source of energy use, gaseous emissions, wastewater, and solid waste.

Human capital is measured as the number of students enrolled in higher education divided by the year-end resident population. This ratio captures the concentration of higher-education students relative to the size of the regional population. A higher value therefore indicates a greater concentration of human capital. Human capital is included because regional educational capacity may influence the adoption of advanced technologies, environmental awareness, administrative capability, and public demand for environmental quality (66, 67).

#### 3.2.5 Other relevant variables

Environmental investment and electricity consumption are used to examine regional heterogeneity in the relationship between artificial intelligence and environmental public health risks.

Environmental investment is measured by the total financial resources allocated to environmental protection, representing a region’s financial capacity for pollution treatment, environmental infrastructure, ecological restoration, and related public programs. It complements the environmental regulation ratio by capturing the absolute level of financial resources devoted to environmental protection. Fiscal environmental expenditure may influence both regional environmental capacity and pollution-related outcomes (68).

Electricity consumption is measured as total regional electricity use, representing the scale and energy intensity of regional economic activity. Higher electricity consumption may indicate larger industrial systems and greater dependence on energy-intensive production. It may also provide greater scope for artificial intelligence to improve load forecasting, equipment scheduling, production coordination, and energy efficiency.

Internet webpages and IPv4 addresses are used as supplementary measures of general digital infrastructure. Internet webpages are measured by the total number of accessible webpages, while IPv4 addresses are measured by the number of allocated internet protocol addresses. These measures reflect internet availability, network carrying capacity, and regional digital connectivity. They are used to distinguish the environmental association of artificial intelligence from the broader effects of basic internet infrastructure (68, 69).

Table 4 reports the definitions, calculation methods, symbols, and data sources for all variables. Table 5 presents the descriptive statistics.

**Table 4:** Description of variables.

Variable	Definition	Calculation methods		Symbol	Data sources
Explained variable	Environmental public health risk	Releases of carbon dioxide		EPHR	EDGAR database
		Sulfur dioxide emissions from industrial sources			The China Environmental Statistical Yearbook; the China City Statistical Yearbook; provincial statistical communiques
		Discharge of wastewater from industrial sources			
		Industrial nitrogen oxide content in the atmosphere			
		Industrial solid waste			
Explanatory variable	Artificial intelligence	Research capability	Number of research and development projects in universities	AI	The China Statistical Yearbook on Science and Technology
			Number of research and development institutions in major industrial enterprises		
			Number of employees in information transmission and software sectors		
		Financial support	Internal expenditure on research and development		
			Funding expenditure of research institutions		
			Internal research and development expenditure in high-technology industries		
		Research development	Transaction turnover in technology markets		
			Number of patent applications by high technology enterprises		
			Scientific papers published by universities		
Mediating variable	Technological expenditure	The aggregate sum of scientific and technical expenditures		TE	The China Statistical Yearbook
	Green patents	The quantity of submissions for green patents		GP	The Chinese Research Data Services Platform
Control variables	Environmental regulation	The proportion of finalized investment in industrial pollution mitigation to industrial value added		ER	The China Statistical Yearbook
	Industrial agglomeration	The proportion of total employees to total land area under administrative control		AGG	
	Industrialization level	The percentage of industrial value added to regional output		IND	
	Human capital	Number of students enrolled in higher education divided by the year-end resident population		HC	
Other relevant variables	Environmental investment	The total financial resources invested in environmental health protection		EI	The China Environmental Statistical Yearbook
	Electricity consumption	The total amount of electricity consumption		EC	China National Bureau of Statistics
	Internet webpages	The sum of all accessible web pages		IW	China Internet Network Information Center; China Statistical Yearbook; provincial Statistical Yearbook
	IPv4 addresses	The sum of allotted IPv4 addresses		IPv4	

**Table 5:** Statistical descriptions of the variables.

Variable	*N*	Min	Median	Max	Mean	SD
EPHR	300	0.93568	1.80348	4.72326	2.00000	0.76603
AI	300	0.06892	0.57815	0.96839	0.55809	0.19099
TE	300	0.00094	0.00886	0.25755	0.02162	0.03041
GP	300	0.00530	0.20440	2.54200	0.40843	0.52967
ER	300	0.00006	0.00186	0.03098	0.00282	0.00336
AGG	300	0.00036	0.01528	0.21707	0.02575	0.03906
IND	300	0.10075	0.31089	0.49756	0.30839	0.07513
HC	300	0.00919	0.02191	0.04374	0.02248	0.00583
EI	300	0.00100	0.02045	0.09525	0.02442	0.01830
EC	300	0.02519	0.18594	0.85020	0.23802	0.17186
IW	300	0.00001	0.00220	0.14090	0.00956	0.02071

### 3.3 Data sources and processing

This study selects a panel dataset comprising 30 provincial regions in China from 2014 to 2023, excluding Tibet, Taiwan, Hong Kong, and Macao. This selection carefully accounts for administrative boundary changes and missing data over the study period.

The primary data sources for constructing the artificial intelligence index (research capability, financial support, and research development) are derived from the China Statistical Yearbook on Science and Technology. Data for technological expenditure and key control variables are obtained from the China Statistical Yearbook. Additionally, data regarding green patents are extracted from the Chinese Research Data Services Platform. Statistics concerning regional EC and environmental investment are sourced from the China National Bureau of Statistics and the China Environmental Statistical Yearbook, respectively. Furthermore, data regarding regional digital infrastructure (internet webpages and IPv4 Addresses) are systematically obtained from the statistical reports of the China Internet Network Information Center, and are further supplemented and cross-referenced by the China Statistical Yearbook as well as various provincial statistical yearbooks.

With the exception of ${\mathrm{CO}}_{2}$ , all raw data for the constituent indicators of environmental public health risks, namely ${\mathrm{SO}}_{2}$ , Water, ${\mathrm{NO}}_{X}$ , and ISW, have been comprehensively sourced from the China Environmental Statistical Yearbook and supplemented by the China City Statistical Yearbook and provincial statistical communiques. Due to the lack of official provincial ${\mathrm{CO}}_{2}$ datasets, this study utilizes the EDGAR database, following the methodology established by Lekaki et al. (2024) (57). Finally, specific volume indicators underwent necessary magnitude standardizations to maximize empirical reliability.

## 4 Empirical results

### 4.1 Pre-estimation diagnostics and baseline regression

#### 4.1.1 Panel unit root and cointegration tests

Before estimating the baseline model, the Levin–Lin–Chu panel unit root test examines the time-series properties of the variables (70). As reported in Table 6, environmental public health risks, artificial intelligence, environmental regulation, industrial agglomeration, and industrialization level are stationary in levels and are therefore classified as *I*(0). Human capital becomes stationary after first differencing and is classified as *I* (1). The variables therefore exhibit mixed orders of integration.

**Table 6:** Results of the LLC panel unit root test.

Variable	Statistic	*p*-value	Result
EPHR	−42.994***	0.000	I(0)
AI	−1.775**	0.038	I(0)
ER	−13.706***	0.000	I(0)
AGG	−1.856**	0.032	I(0)
IND	−6.900***	0.000	I(0)
HC	−6.766***	0.000	I(1)

***, **, and * indicate statistical significance at the 1, 5, and 10% levels, respectively. Variables that are stationary in levels are denoted as I(0), while variables that become stationary after first differencing are denoted as I(1).

Fixed-effects estimation does not require all variables to follow the same non-stationary process. The Kao residual-based cointegration test is nevertheless reported as a supplementary diagnostic (71). As shown in Table 7, four of the five reported statistics reject the null hypothesis of no cointegration at the 1% or 5% level, while the augmented Dickey–Fuller statistic is not statistically significant. These results provide supplementary evidence consistent with a stable long-run relationship among the variables. Because the variables exhibit mixed orders of integration, the Kao results are interpreted cautiously and are not used as the primary justification for the two-way fixed-effects model.

**Table 7:** Supplementary Kao panel cointegration test results.

Test statistic	Value	*p*-value
Modified Dickey–Fuller t	−2.255**	0.012
Dickey–Fuller t	−10.939***	0.000
Augmented Dickey–Fuller t	−0.715	0.237
Unadjusted modified Dickey–Fuller t	−4.865***	0.000
Unadjusted Dickey–Fuller *t*	−12.09***	0.000

***, **, and * indicate statistical significance at the 1, 5, and 10% levels, respectively. The null hypothesis is no cointegration. The Kao test is reported as a supplementary diagnostic.

#### 4.1.2 Baseline regression results

Table 8 reports the estimated association between artificial intelligence and environmental public health risks. Column (1) includes artificial intelligence without control variables, while column (2) adds environmental regulation, industrial agglomeration, industrialization level, and human capital. Provincial and year fixed effects are included in both specifications.

**Table 8:** Baseline two-way fixed-effects estimates of artificial intelligence and environmental public health risks.

Variable	(1)	(2)
	EPHR	EPHR
AI	−0.967** (0.470)	−1.109** (0.509)
ER		−7.693* (4.578)
AGG		6.562 (8.273)
IND		−0.299 (0.353)
HC		−8.108 (7.322)
Cons	2.539*** (0.262)	2.746*** (0.416)
Provincial region	Yes	Yes
Year	Yes	Yes
R^2^	0.967	0.968
*N*	300	300

Standard errors are reported in parentheses. All models include provincial and year fixed effects. Column (2) controls for environmental regulation, industrial agglomeration, industrialization level, and human capital. ***, **, and * indicate statistical significance at the 1, 5, and 10% levels, respectively.

The coefficient of artificial intelligence is negative and statistically significant at the 5% level in both models. In column (1), the coefficient is (−0.967), indicating that a 0.01-unit increase in the artificial intelligence index is associated with a 0.00967-unit decrease in the environmental public health risk index. After the control variables are included, the coefficient is (−1.109). A 0.01-unit increase in the artificial intelligence index is therefore associated with a 0.01109-unit decrease in the environmental public health risk index, holding the included controls and fixed effects constant.

The stability of the coefficient across the two specifications indicates that the negative association is not driven solely by the included regional economic and environmental controls. Environmental regulation also has a negative coefficient in column (2), although it is statistically significant only at the 10% level. The coefficients of industrial agglomeration, industrialization level, and human capital are not statistically significant in the baseline specification. These results support Hypothesis 1.

#### 4.1.3 Cross-sectional dependence test

A Pesaran cross-sectional dependence test is conducted on the residuals from the baseline two-way fixed-effects model. This test evaluates whether unexplained correlations remain across provincial regions after including control variables and fixed effects.

As reported in Table 9, the cross-sectional dependence statistic is 0.570, with a *p*-value of 0.572. The null hypothesis of cross-sectional independence cannot be rejected. Therefore, the test does not provide evidence of residual cross-sectional dependence in the estimated baseline model. This result supports using the reported fixed-effects specification, though it does not establish that all possible spatial relationships or common shocks have been eliminated.

**Table 9:** Cross-sectional dependence test on baseline model residuals.

Variable	CD-test statistic	*p*-value	corr	abs(corr)
Baseline residuals	0.570	0.572	0.009	0.634

The null hypothesis is cross-sectional independence. The test is applied to the residuals from the baseline two-way fixed-effects model.

### 4.2 Mechanism analysis

The proposed mediation pathways are evaluated in two stages. Table 10 reports the component paths estimated using models with provincial and year fixed effects, while Table 11 evaluates the corresponding indirect effects using 5,000 province-clustered Bootstrap resamples. The fixed-effects component paths indicate whether artificial intelligence is related to each proposed mediator and whether the mediator is associated with environmental public health risks after controlling for artificial intelligence. However, mediation is primarily assessed from the Bootstrap confidence intervals for the product of the two component coefficients.

**Table 10:** Two-way fixed-effects estimates of the proposed mediation pathways.

Variable	(1)	(2)	(3)	(4)	(5)
	EPHR	TE	EPHR	GP	EPHR
GP					−0.183*** (0.066)
TE			−0.884* (0.490)		
AI	−1.109** (0.509)	0.156** (0.065)	−0.971* (0.513)	1.411*** (0.478)	−0.851* (0.511)
ER	−7.693* (4.578)	0.750 (0.581)	−7.030 (4.573)	15.082*** (4.300)	−4.935 (4.626)
AGG	6.562 (8.273)	−0.053 (1.051)	6.515 (8.237)	31.064*** (7.771)	12.243 (8.417)
IND	−0.299 (0.353)	0.000 (0.045)	−0.299 (0.351)	−0.543 (0.331)	−0.398 (0.350)
HC	−8.108 (7.322)	−2.456*** (0.930)	−10.279 (7.389)	−57.730*** (6.878)	−18.666** (8.162)
Cons	2.746*** (0.416)	−0.011 (0.053)	2.736*** (0.414)	0.243 (0.391)	2.791*** (0.411)
Provincial region	Yes	Yes	Yes	Yes	Yes
Year	Yes	Yes	Yes	Yes	Yes
R^2^	0.968	0.667	0.968	0.940	0.968
*N*	300	300	300	300	300

Standard errors are reported in parentheses. All models include provincial and year fixed effects and control for environmental regulation, industrial agglomeration, industrialization level, and human capital. Column (1) reports the baseline association. Columns (2) and (3) examine the technological expenditure pathway, while columns (4) and (5) examine the green patent pathway. The statistical significance of the indirect effects is evaluated separately using the province-clustered Bootstrap confidence intervals reported in Table 11. ***, **, and * indicate statistical significance at the 1, 5, and 10% levels, respectively.

**Table 11:** Province-clustered Bootstrap tests of indirect effects.

Hypothesis	Mediating path	Indirect effect	Bootstrapped std. err.	Percentile 95% CI	95% confidence interval (BC)	Result
H2a	AI → TE → EPHR	−0.137	0.085	[−0.310, 0.005]	[−0.475, −0.026]	Qualified support
H2b	AI → GP → EPHR	−0.258	0.329	[−1.074, 0.154]	[−1.074, 0.153]	No supported

The indirect effects are estimated using 5,000 province-clustered Bootstrap resamples. All component equations include provincial and year fixed effects and control for environmental regulation, industrial agglomeration, industrialization level, and human capital. Percentile and bias-corrected and accelerated (BCa) 95% confidence intervals are reported. TE denotes technological expenditure, GP denotes green patents, AI denotes artificial intelligence, and EPHR denotes environmental public health risks.

#### 4.2.1 Mediating role of technological expenditure

Technological expenditure may provide an implementation channel through which artificial intelligence translates into environmental action. Intelligent monitoring, predictive analysis, and environmental data integration require ongoing expenditure on sensors, computing infrastructure, software, technical personnel, cleaner equipment, and energy management systems. Artificial intelligence may also improve the allocation of technological resources by helping regional authorities and firms identify pollution-intensive activities and prioritize projects with greater environmental value (72, 73).

The fixed-effects estimates in Table 10 provide initial evidence consistent with this pathway. In column (2), artificial intelligence is positively associated with technological expenditure (*β* = 0.156, SE = 0.065, *p* < 0.05). In column (3), technological expenditure is negatively associated with environmental public health risks after including artificial intelligence and the baseline controls (*β* = −0.884, SE = 0.490, *p* < 0.10). The coefficient of artificial intelligence decreases in absolute magnitude from (−1.109) in the baseline model to (−0.971) after technological expenditure is introduced. The corresponding product-of-coefficients estimate is approximately (−0.137).

These component estimates align with the possibility that artificial intelligence supports environmental risk reduction partly by facilitating technological investment and implementation. Nevertheless, the statistical significance of individual paths does not, by itself, establish a stable indirect effect. Table 11, therefore, reports the province-clustered Bootstrap inference. The estimated indirect effect through technological expenditure is (−0.137), with a Bootstrap standard error of 0.085. The percentile 95% confidence interval ranges from (−0.310) to (0.005) and thus includes zero, whereas the bias-corrected and accelerated 95% confidence interval ranges from (−0.475) to (−0.026) and excludes zero.

The direction of the indirect effect is consistent across the fixed-effects and Bootstrap estimates, but its statistical conclusion depends on the confidence interval procedure. Hypothesis 2a receives qualified support.

#### 4.2.2 Mediating role of green patents

Green patents represent formalized outputs of environmentally oriented innovation. Artificial intelligence may support green patent development by improving technological search, reducing experimentation costs, and accelerating the identification of solutions related to cleaner manufacturing, energy efficiency, and pollution treatment (74, 75). However, patent applications do not necessarily generate immediate environmental improvements. Their practical value depends on patent quality, granting, commercialization, industrial adoption, and diffusion into pollution-intensive activities.

The fixed-effects component paths in Table 10 are consistent with the proposed mechanism. In column (4), artificial intelligence is positively associated with green patent applications (*β* = 1.411, SE = 0.478, *p* < 0.01). In column (5), green patent applications are negatively associated with environmental public health risks after artificial intelligence and the remaining covariates are included ((\beta = −0.183), (SE = 0.066), (*p* < 0.01)). The coefficient of artificial intelligence decreases in absolute magnitude from (−1.109) in the baseline model to (−0.851) after green patents are introduced. The corresponding product-of-coefficients estimate is approximately (−0.258).

The estimated indirect effect is (−0.258), with a Bootstrap standard error of 0.329. The percentile 95% confidence interval ranges from (−1.074) to (0.154), and the bias-corrected and accelerated 95% confidence interval ranges from (−1.074) to (0.153). Both intervals include zero.

The significant component paths indicate that artificial intelligence development, green patent activity, and environmental public health risks are related in the directions proposed by the conceptual model. However, their product is not estimated with sufficient precision after provincial characteristics, common annual shocks, and within-province dependence are taken into account. Hypothesis 2b is therefore not supported by the main Bootstrap analysis.

The difference between the two proposed mediators is substantively informative. Technological expenditure is an input that can be used directly to deploy monitoring equipment, data systems, cleaner machinery, and technical personnel within the study period. Green patent applications represent an earlier and less immediate stage of the innovation process. Their environmental effects may require additional time and successful conversion into commercially and operationally adopted technologies. The pooled mediation estimates reported in Appendix A show significant indirect effects through both variables, but those estimates do not control for provincial and year fixed effects. The contrast suggests that the pooled green patent result may reflect persistent differences in regional innovation capacity more strongly than a stable short-term within-province mediation process.

### 4.3 Heterogeneity analysis

Table 12 examines whether environmental investment and electricity consumption change the association between artificial intelligence and environmental public health risks. Both models include the corresponding regional variable, its interaction with artificial intelligence, the baseline control variables, and provincial and year fixed effects.

**Table 12:** Heterogeneity and marginal-effect results.

(A) Interaction estimates		
Variable	(1) Environmental investment	(2) Electricity consumption
AI	−0.843 (0.522)	0.434 (0.458)
EI	13.889*** (3.511)	
AI × EI	−16.434*** (5.213)	
EC		−0.829 (0.757)
AI × EC		−1.897*** (0.715)
ER	−9.469** (4.451)	−2.910 (3.840)
AGG	3.574 (8.067)	19.000*** (6.936)
IND	0.030 (0.350)	−0.487 (0.301)
HC	−9.597 (7.454)	−24.112*** (6.490)
Provincial fixed effects	Yes	Yes
Year fixed effects	Yes	Yes
R^2^	0.970	0.978
*N*	300	300

(B) Marginal effects of artificial intelligence at representative regional conditions						
Regional condition	Level	Value	Marginal effect of AI	SE	*p*-value	95% CI
Environmental investment	25th percentile	0.0105	−1.015	0.508	0.047	[−2.016, −0.015]
Environmental investment	Median	0.0205	−1.179	0.501	0.019	[−2.165, −0.193]
Environmental investment	75th percentile	0.0332	−1.388	0.499	0.006	[−2.370, −0.407]
Electricity consumption	25th percentile	0.1166	0.213	0.439	0.628	[−0.651, 1.078]
Electricity consumption	Median	0.1859	0.082	0.435	0.851	[−0.775, 0.939]
Electricity consumption	75th percentile	0.2905	−0.117	0.440	0.791	[−0.983, 0.750]

Standard errors are reported in parentheses. Both models include provincial and year fixed effects and control for environmental regulation, industrial agglomeration, industrialization level, and human capital. ***, **, and * indicate statistical significance at the 1, 5, and 10% levels, respectively.

The interaction between artificial intelligence and environmental investment is negative and statistically significant at the 1% level (*β* = −16.434, *p* < 0.01). The marginal-effect estimates provide a clearer interpretation of this interaction. At the 25th percentile of environmental investment, the estimated coefficient of artificial intelligence is (−1.015) (*p* = 0.047). The coefficient becomes (−1.179) at the median (*p* = 0.019) and (−1.388) at the 75th percentile (*p* = 0.006). These estimates indicate that artificial intelligence is associated with lower environmental public health risks across representative levels of environmental investment and that the negative association strengthens as environmental investment increases. Greater environmental investment may improve provincial regions’ capacity to convert digital capability into environmental monitoring, pollution treatment equipment, and related infrastructure. Hypothesis 3a is therefore supported.

The interaction between artificial intelligence and electricity consumption is also negative and statistically significant (*β* = −1.897, *p* < 0.01). This result indicates that the slope of artificial intelligence becomes more negative as electricity consumption increases. The marginal effects of artificial intelligence at the 25th percentile, median, and 75th percentile of electricity consumption are 0.213, 0.082, and (−0.117), respectively. None of these conditional estimates are statistically different from zero. The interaction therefore provides evidence that electricity consumption changes the direction and magnitude of the artificial intelligence coefficient, although it does not establish a statistically significant negative association at the representative levels examined. Hypothesis 3b receives qualified support.

### 4.4 Robustness analyses

Several supplementary analyses assess whether the baseline association is sensitive to alternative measurement, additional digital-infrastructure controls, outlier treatment, and concurrent national policies.

#### 4.4.1 Dynamic persistence and system GMM sensitivity analysis

Environmental public health risks can persist over time due to the gradual adjustment of industrial structures, accumulated pollution, environmental infrastructure, and regulatory capacity. The baseline relationship may also be affected by reverse causality or omitted time-varying factors, as regions with greater environmental pressure may simultaneously increase their investment in artificial intelligence. Therefore, a System Generalized Method of Moments model is estimated as a supplementary dynamic analysis. This model incorporates the first-order lag of the dependent variable and uses internal instruments to address potential dynamic panel bias and endogeneity concerns (76, 77).

The model is estimated using a two-step System GMM procedure with corrected standard errors. Environmental public health risks and artificial intelligence are treated as endogenous variables. The second lag of environmental public health risks and the second and third lags of artificial intelligence are used as GMM-style instruments. Environmental regulation, industrial agglomeration, industrialization level, and human capital are included as IV-style variables. Year effects are incorporated, and the instrument matrix is collapsed to limit instrument proliferation.

As reported in Table 13, the coefficient of the first-order lag of environmental public health risks is positive and statistically significant at the 1% level ((\beta = 0.872), (SE = 0.080), (*p* < 0.001)). This result indicates strong temporal persistence in pollution-related environmental conditions. Provinces with relatively high environmental public health risks in one year tend to retain elevated risk levels in the following year, suggesting that accumulated environmental pressure cannot be reversed immediately.

**Table 13:** System GMM estimates accounting for dynamic persistence and potential endogeneity.

Variable	Coefficient	Corrected SE	*p*-value
*L1. EPHR*	0.872***	0.080	<0.001
AI	0.626*	0.340	0.065
ER	9.407*	5.173	0.069
AGG	−1.361	0.984	0.167
IND	−0.112	0.305	0.713
HC	−5.246	5.529	0.343
Constant	0.008	0.121	0.944
Year effects	Yes		
Observations	240		
Provincial groups	30		
Number of instruments	18		
AR(1) test *p*-value	0.002		
AR(2) test *p*-value	0.788		
Hansen test *p*-value	0.110		

The table reports two-step System GMM estimates with corrected standard errors. Environmental public health risks and artificial intelligence are treated as endogenous variables. The second lag of environmental public health risks and the second and third lags of artificial intelligence are used as GMM-style instruments. Environmental regulation, industrial agglomeration, industrialization level, and human capital are entered as IV-style variables. Year effects are included, and the instrument matrix is collapsed. AR(1) and AR(2) are Arellano–Bond tests for first- and second-order serial correlation in the differenced residuals. The Hansen test evaluates the joint validity of the instrument set. The model contains 18 instruments and 30 provincial groups. ***, **, and * indicate statistical significance at the 1, 5, and 10% levels, respectively.

The contemporaneous coefficient of artificial intelligence is positive and marginally significant at the 10% level ((\beta = 0.626), (SE = 0.340), (*p* = 0.065)). This estimate differs from the negative coefficient obtained from the principal two-way fixed-effects models. The System GMM result therefore does not independently confirm the negative contemporaneous association reported in the baseline analysis. Instead, it indicates that the estimated relationship is sensitive to the treatment of temporal persistence, endogenous regressors, and internal instruments.

One possible interpretation is that the environmental costs and benefits of artificial intelligence emerge at different stages. The expansion of computing infrastructure, data centers, intelligent equipment, and organizational capacity may initially increase energy use and implementation costs. In contrast, improvements in pollution monitoring, production efficiency, energy scheduling, and cleaner operations may require a longer period to materialize. Under this interpretation, the positive contemporaneous coefficient may reflect short-run deployment and adjustment pressures, while the negative fixed-effects association may capture broader within-province improvements that develop over time.

The remaining control variables provide limited additional evidence in the dynamic specification. Environmental regulation has a positive coefficient that is marginally significant at the 10% level ((\beta = 9.407), (*p* = 0.069)), while the coefficients of industrial agglomeration, industrialization level, and human capital are not statistically significant.

The Arellano–Bond AR (1) test is statistically significant (*p* = 0.002), which is expected for first-differenced residuals. The AR (2) test is not statistically significant (*p* = 0.788), providing no evidence of second-order serial correlation. The Hansen test yields a *p*-value of 0.110 and therefore does not reject the joint validity of the instrument set at conventional significance levels. The model uses 18 instruments for 30 provincial groups, so the number of instruments remains below the number of cross-sectional units.

#### 4.4.2 Alternative measurement of environmental public health risks

The baseline environmental public health risk index is constructed using the principal component factor method. To examine whether the estimated relationship depends on this particular aggregation procedure, an alternative index is constructed from the same five pollution-related indicators using the entropy-weight method. Industrial wastewater discharge, carbon dioxide emissions, nitrogen oxide emissions, sulfur dioxide emissions, and industrial solid waste are first normalized using positive min–max scaling. Their entropy weights are 0.286851, 0.134559, 0.169895, 0.249974, and 0.158721, respectively. The weighted normalized indicators are then aggregated to form the alternative environmental public health risk index. Column (1) of Table 14 reports the model without the baseline control variables. The coefficient of artificial intelligence is negative and statistically significant at the 1% level (*β* = −0.430, SE = 0.155, *p* < 0.01). After environmental regulation, industrial agglomeration, industrialization level, and human capital are included in column (2), the coefficient remains negative and statistically significant at the 5% level (*β* = −0.425, SE = 0.167, *p* < 0.05).

**Table 14:** Robustness checks using an alternative environmental public health risk measure, additional digital-infrastructure controls, and winsorization.

Variable	(1) Alternative measure	(2) Alternative measure	(3) Baseline controls	(4) Digital controls	(5) Winsorized	(6) Winsorized
AI	−0.430*** (0.155)	−0.425** (0.167)	−1.109** (0.509)	−1.021** (0.512)	−1.156*** (0.427)	−1.203** (0.469)
ER		−1.425 (1.502)	−7.694* (4.578)	−7.710* (4.600)		−9.353* (5.474)
AGG		−1.512 (2.714)	6.562 (8.273)	6.800 (8.410)		4.237 (7.634)
IND		−0.072 (0.116)	−0.299 (0.353)	−0.198 (0.361)		−0.340 (0.343)
HC		−5.518** (2.402)	−8.107 (7.322)	−4.554 (8.358)		−10.039 (6.711)
IW				3.237 (2.229)		
IPv4				−3.624 (4.149)		
Constant	0.433*** (0.086)	0.619*** (0.136)	2.746*** (0.416)	2.921*** (0.586)	2.643*** (0.239)	2.916*** (0.383)
Provincial fixed effects	Yes	Yes	Yes	Yes	Yes	Yes
Year fixed effects	Yes	Yes	Yes	Yes	Yes	Yes
(R^2)	0.907	0.909	0.968	0.968	0.971	0.972
(N)	300	300	300	300	300	300

Standard errors are reported in parentheses. Columns (1) and (2) use an entropy-weighted environmental public health risk index as the dependent variable. Column (3) reports the baseline specification with the original control variables, while column (4) additionally controls for internet webpages and IPv4 addresses. Columns (5) and (6) use continuous variables winsorized at the 1st and 99th percentiles. All specifications include provincial and year fixed effects. ***, **, and * indicate statistical significance at the 1, 5, and 10% levels, respectively.

Because artificial intelligence is measured as a level index rather than a logarithmic variable, a 0.01-unit increase in the artificial intelligence index is associated with decreases of approximately 0.00430 and 0.00425 units in the alternative environmental public health risk index in columns (1) and (2), respectively. The preservation of both the negative direction and statistical significance indicates that the baseline relationship is not dependent solely on the principal component factor weighting procedure.

#### 4.4.3 Additional digital-infrastructure controls

Regional artificial intelligence development may be associated with broader digital infrastructure that also affects environmental monitoring, information transmission, and production efficiency. Omitting these underlying conditions could cause the artificial intelligence coefficient to partly capture differences in general digital connectivity. Internet webpages and IPv4 addresses are therefore added to the baseline model as additional controls.

Column (3) of Table 14 reproduces the baseline specification with the original control variables. The coefficient of artificial intelligence is (−1.109) and is statistically significant at the 5% level. When internet webpages and IPv4 addresses are included in column (4), the coefficient changes only modestly to (−1.021) and remains statistically significant at the 5% level (SE = 0.512, *p* = 0.047). The coefficients of internet webpages and IPv4 addresses are not statistically significant.

The continued negative association after including these variables indicates that the artificial intelligence coefficient is not explained solely by broader regional internet infrastructure. Artificial intelligence, therefore, appears to capture technological capabilities beyond basic digital connectivity.

#### 4.4.4 Winsorization of continuous variables

Extreme observations may disproportionately influence estimates obtained from a provincial panel. To assess this possibility, all continuous variables used in the corresponding models are winsorized at the 1st and 99th percentiles, and the fixed-effects models are re-estimated using the adjusted data.

In the specification without the baseline control variables, the coefficient of artificial intelligence is negative and statistically significant at the 1% level (*β* = −1.156, SE = 0.427, *p* < 0.01). After the control variables are included, the coefficient remains negative and statistically significant at the 5% level (*β* = −1.203, SE = 0.469, *p* < 0.05).

The magnitude and direction of these estimates are broadly consistent with the baseline results. The negative association between artificial intelligence and environmental public health risks is therefore not attributable to a small number of extreme observations.

#### 4.4.5 Controlling for concurrent National Policies

Two policy indicators are added separately to the baseline model to account for institutional changes during the sample period. These indicators are treated as additional controls and not as independent quasi-natural experiments.

After the National Big Data Comprehensive Pilot Zone indicator is included, the coefficient of artificial intelligence remains negative and statistically significant at the 5% level (*β* = −1.037, *p* < 0.05). The policy indicator itself has a negative coefficient (*β* = −0.142, *p* < 0.01), indicating that the pilot policy is associated with lower environmental public health risks after the remaining covariates and fixed effects are controlled.

After the environmental vertical management reform indicator is introduced, the coefficient of artificial intelligence also remains negative and statistically significant at the 5% level (*β* = −1.108, *p* < 0.05). The reform indicator is not statistically significant. These results show that the baseline association is not eliminated by the inclusion of the two concurrent policy controls. They do not, however, establish that the artificial intelligence coefficient represents a strictly causal effect (Table 15).

**Table 15:** Results after controlling for concurrent national policies.

Variable	(1) Big-data pilot policy	(2) Environmental management reform
AI	−1.037** (0.501)	−1.108** (0.510)
ER	−6.268 (4.525)	−7.669* (4.587)
AGG	5.659 (8.141)	6.606 (8.289)
IND	−0.154 (0.350)	−0.299 (0.353)
HC	−9.467 (7.214)	−8.119 (7.335)
Policy-BD	−0.142*** (0.045)	
Policy-EM		0.017 (0.053)
Provincial fixed effects	Yes	Yes
Year fixed effects	Yes	Yes
(R^2)	0.969	0.968
(N)	300	300

Standard errors are reported in parentheses. Both specifications include provincial and year fixed effects and baseline control variables. Policy-BD denotes the National Big Data Comprehensive Pilot Zone policy, and Policy-EM denotes environmental vertical management reform. The policy indicators are included as additional controls and are not treated as quasi-natural experiments. ***, **, and * indicate statistical significance at the 1, 5, and 10% levels, respectively.

### 4.5 Non-linear association across environmental regulation levels

The association between artificial intelligence and environmental public health risks may change with the intensity of environmental regulation. Environmental regulation can create demand for intelligent monitoring, compliance assessment, and cleaner production. However, regulation may also directly reduce pollution-related risks through mandatory standards and enforcement, thereby decreasing the additional contribution of artificial intelligence once regulatory intensity reaches a sufficiently high level. Environmental regulation is therefore introduced as the threshold variable in a fixed-effects panel threshold model (78, 79).

The threshold model treats artificial intelligence as the regime-dependent variable and controls for environmental regulation, industrial agglomeration, industrialization level, and human capital. The threshold search applies a 1% trimming parameter and a grid size of 100. Threshold significance is evaluated using 1,000 Bootstrap replications.

As reported in Table 16, the single-threshold test produces an F-statistic of 26.83 and a Bootstrap *p*-value of 0.035. The estimated threshold is 0.003784, reported as 0.0038 after rounding, and its 95% confidence interval ranges from 0.0035 to 0.0039. The double- and triple-threshold tests are not statistically significant. The results therefore support a single change in the coefficient of artificial intelligence across environmental regulation levels.

**Table 16:** Bootstrap test for the environmental regulation threshold.

Threshold variable	Threshold specification	Threshold estimate	95% confidence interval	F-statistic	Bootstrap *p*-value	10% critical value	5% critical value	1% critical value
Environmental regulation	Single threshold	0.0038	[0.0035, 0.0039]	26.83	0.035**	20.716	25.642	35.581

Threshold significance is evaluated using 1,000 Bootstrap replications, a 1% trimming parameter, and a grid size of 100. The null hypothesis is that no threshold effect exists. Double- and triple-threshold tests are not statistically significant and are therefore not reported in detail. ***, **, and * indicate statistical significance at the 1, 5, and 10% levels, respectively.

Table 17 presents the estimates from the supported single-threshold model. When environmental regulation is at or below 0.0038, the coefficient of artificial intelligence is negative and statistically significant at the 1% level (*β* = −2.293, SE = 0.656, *p* < 0.01). When environmental regulation exceeds 0.0038, the coefficient remains negative and statistically significant at the 1% level, but its absolute magnitude decreases to 1.923 (*β* = −1.923, SE = 0.633, *p* < 0.01).

**Table 17:** Fixed-effects panel threshold estimates with environmental regulation as the threshold variable.

Variable	Single-threshold model
Environmental regulation	8.599 (6.635)
Industrial agglomeration	−1.497 (12.598)
Industrialization level	1.239** (0.538)
Human capital	−8.942 (9.106)
AI × I(ER ≤ 0.0038)	−2.293*** (0.656)
AI × I(ER > 0.0038)	−1.923*** (0.633)
Constant	3.077*** (0.489)
Provincial fixed effects	Yes
Within R^2^	0.541
Observations	300

Province-clustered robust standard errors are reported in parentheses. Artificial intelligence is the regime-dependent variable, and environmental regulation is the threshold variable. The lower regulatory regime is defined as ER ≤ 0.0038, while the upper regulatory regime is defined as ER > 0.0038. ***, **, and * indicate statistical significance at the 1, 5, and 10% levels, respectively.

Artificial intelligence is therefore associated with lower environmental public health risks in both regulatory regimes. However, the absolute magnitude of the coefficient decreases by approximately 16.1% after environmental regulation crosses the estimated threshold. The result reveals a diminishing marginal pattern rather than an activation effect: Stronger regulation does not eliminate the negative association of artificial intelligence with environmental public health risks, but it reduces its additional magnitude.

One possible explanation is partial substitution between formal regulation and technology-enabled environmental management. When regulation is comparatively limited, artificial intelligence-based monitoring, pollution detection, energy optimization, and production scheduling may provide a relatively large additional contribution to environmental risk reduction. Once regulation becomes stronger, mandatory standards, regulatory inspections, and compliance investments may already produce part of the attainable improvement, leaving less incremental scope for artificial intelligence. This interpretation does not imply that regulation and artificial intelligence are incompatible. It suggests that their relative contributions change as the regulatory environment develops.

The evidence supports Hypothesis 4, demonstrating that the association between artificial intelligence and environmental public health risks varies with environmental regulation levels. Specifically, the negative association persists across both sides of the threshold but weakens in absolute magnitude above the estimated regulatory level.

## 5 Discussion

The main results indicate that artificial intelligence is associated with lower pollution-related environmental public health risks in the two-way fixed-effects models. This relationship aligns with the operational functions through which intelligent systems can contribute to environmental management. Artificial intelligence can integrate information from monitoring networks, identify abnormal emission patterns, improve pollution forecasting, and shorten the time between risk detection and administrative or industrial response (19–21, 80, 81). It can also support energy scheduling, predictive maintenance, transportation optimization, and cleaner production decisions, thereby reducing avoidable resource use and hazardous emissions (22, 23, 32, 82). The alternative-index model, additional digital-infrastructure controls, winsorization, and concurrent policy controls preserve the negative direction of the baseline estimate. The dynamic analysis, however, adds an important qualification. Environmental public health risks exhibit strong temporal persistence in the System GMM model, while the contemporaneous artificial intelligence coefficient becomes positive and is significant only at the 10% level. This difference indicates that the estimated relationship is sensitive to the treatment of dynamic persistence and internal instruments. One possible explanation is that the short-run energy, infrastructure, and adjustment costs associated with artificial intelligence development may arise before its monitoring and efficiency benefits are fully realized (4–7, 13).

The comparison between technological expenditure and green patents clarifies how the association with artificial intelligence may develop. Technological expenditure offers limited but meaningful evidence of an indirect pathway. Artificial intelligence requires ongoing investment in monitoring equipment, computing infrastructure, software, technical personnel, and production system integration. When these resources are directed toward sensors, pollution treatment equipment, cleaner production systems, and energy management platforms, digital capacity can translate into operational environmental improvements (27–32). The province-clustered Bootstrap result, however, shows that this indirect effect is estimated with limited precision, so technological expenditure should be regarded as a plausible implementation channel rather than a fully established mechanism. The evidence for green patents is even more dependent on model specification. The indirect effect through green patents is significant in the pooled model but is no longer statistically stable after accounting for provincial characteristics, common annual shocks, and within-province dependence. This contrast suggests that green patents may primarily capture persistent differences in regional innovation capacity. Provinces with stronger artificial intelligence systems may also maintain larger green patent portfolios and lower pollution-related risks over long periods, but annual increases in green patent applications do not necessarily produce an immediate within-province reduction during the sample period. Patent applications represent codified innovation outputs, while their environmental value depends on quality, implementation, commercialization, and adoption by pollution-intensive industries (33–37, 74, 75). Technological expenditure and green patents therefore occupy different positions in the innovation process: Expenditure can support current deployment, whereas the benefits of patented technologies may emerge only after a longer and less certain conversion process.

The heterogeneity results show that regional resources and production conditions do not influence the artificial intelligence relationship in the same way. Environmental investment produces a clearer boundary condition. The interaction is significantly negative, and the marginal effects of artificial intelligence remain negative and statistically significant at the 25th percentile, median, and 75th percentile of environmental investment. Moreover, the negative coefficient becomes larger in absolute magnitude as environmental investment increases. This pattern indicates that financial and infrastructural support helps transform artificial intelligence from a general digital capability into an operational environmental management resource. Environmental investment can finance monitoring networks, pollution treatment facilities, ecological restoration, equipment replacement, and the integration of intelligent systems into traditional industrial processes (29, 31, 40–44, 68).

Electricity consumption presents a more cautious result. The negative interaction indicates that the slope of artificial intelligence becomes more negative as electricity consumption rises, which is compatible with the idea that larger energy systems offer broader opportunities for load forecasting, equipment optimization, and the reduction of operational inefficiency (32, 45–48). However, the conditional artificial intelligence coefficients at the representative electricity consumption levels are not statistically different from zero. Higher electricity consumption may therefore create a larger potential field for optimization, but it also represents energy dependence and pollution pressure rather than an environmental advantage in itself (83). Environmental investment provides direct implementation capacity, whereas electricity consumption mainly identifies the scale of the technical challenge and the possible scope for optimization.

The environmental regulation threshold reveals a diminishing marginal pattern. Artificial intelligence remains negatively and significantly associated with environmental public health risks in both regulatory regimes. When environmental regulation is at or below the estimated threshold of 0.0038, the artificial intelligence coefficient is −2.293. Above the threshold, the coefficient remains negative but decreases in absolute magnitude to −1.923. The additional negative association attributed to artificial intelligence becomes smaller once regulation exceeds the threshold. This pattern may reflect partial substitution between formal regulation and technology-enabled environmental management. Under comparatively limited regulation, intelligent monitoring, pollution detection, and production optimization may substantially augment existing environmental controls. Once regulation becomes stronger, mandatory emission standards, inspections, compliance investments, and pollution treatment requirements may already achieve part of the attainable improvement, leaving less incremental scope for artificial intelligence. It may also reflect diminishing marginal returns when regulatory and technological instruments address overlapping sources of environmental inefficiency. Threshold effects in environmental regulation have similarly been associated with changes in the marginal influence of economic and technological factors across regulatory regimes (78, 79). The result therefore supports coordinated policy design: Environmental regulation and artificial intelligence remain complementary components of risk management, but continually increasing regulatory intensity should not be assumed to magnify the marginal contribution of artificial intelligence.

## 6 Conclusion and policy implications

### 6.1 Conclusion

This study demonstrates that artificial intelligence has meaningful potential to support the reduction of pollution-related environmental public health risks, but that this potential depends on how digital capability is translated into environmental action. Using provincial panel data from 2014 to 2023 and a multidimensional index covering gaseous emissions, industrial wastewater, and industrial solid waste, the analysis shows that higher levels of artificial intelligence are associated with lower environmental public health risks in the principal two-way fixed-effects models. This negative relationship remains under an alternative entropy-weighted risk index, additional digital-infrastructure controls, winsorization, and controls for concurrent national policies.

The findings suggest that the value of artificial intelligence lies in its capacity to improve the operation of environmental and industrial systems. Intelligent monitoring can identify abnormal emissions more quickly, predictive models can anticipate changes in pollution conditions, and data-driven production and energy management can reduce avoidable resource use and operational waste. Artificial intelligence should therefore be understood not merely as a digital technology, but as an enabling capability that can strengthen the speed, precision, and coordination of pollution-related risk management.

The dynamic analysis adds a temporal qualification. Environmental public health risks display strong persistence, while the contemporaneous artificial intelligence coefficient changes direction in the System GMM specification and is estimated only at the 10% significance level. This result indicates that the environmental contribution of artificial intelligence may not emerge immediately. The construction of data infrastructure, computing capacity, intelligent equipment, and organizational expertise can initially require substantial energy and financial resources, whereas improvements in monitoring, production efficiency, and pollution control may develop over a longer period. The difference between the fixed-effects and dynamic estimates therefore highlights a possible timing gap between the costs of artificial intelligence deployment and the realization of its environmental benefits.

The mechanism analysis further shows that the environmental contribution of artificial intelligence depends on implementation capacity. Technological expenditure provides qualified evidence of an indirect pathway because it can convert digital capability into sensors, pollution treatment equipment, software systems, technical personnel, and energy management infrastructure. Green patents show a different pattern. Their indirect effect is visible in the pooled analysis but becomes statistically unstable after provincial and annual differences are controlled. This result does not imply that green innovation is unimportant. Instead, it suggests that patent applications reflect a longer-term regional innovation capacity whose environmental benefits depend on subsequent quality, commercialization, industrial adoption, and diffusion. Artificial intelligence may therefore influence environmental public health risks through channels operating at different speeds: Technological expenditure supports current deployment, whereas green patents may contribute through a slower innovation conversion process.

Regional conditions also determine whether artificial intelligence can be translated into environmental improvement. Environmental investment provides the clearest supporting condition. The negative association of artificial intelligence becomes stronger as environmental investment increases, and its marginal effects remain significant across low, median, and high representative investment levels. This finding indicates that intelligent technologies require complementary financial and physical infrastructure. Electricity consumption provides a more conditional boundary. Higher electricity use increases the potential scope for load forecasting, equipment optimization, and energy efficiency improvement, but the conditional effects of artificial intelligence are not significant at the representative electricity consumption levels. High energy demand therefore creates an opportunity for intelligent optimization, but does not guarantee environmental improvement without cleaner energy structures, suitable infrastructure, and effective implementation.

Environmental regulation introduces a further non-linear condition. Artificial intelligence remains negatively associated with environmental public health risks below and above the estimated regulatory threshold, but the absolute magnitude of the coefficient becomes smaller in the higher-regulation regime. This pattern indicates that artificial intelligence and formal regulation may partly address the same sources of environmental inefficiency. When regulation is relatively limited, intelligent monitoring and production optimization can make a larger additional contribution. Under stronger regulation, mandatory standards, inspections, and compliance investments may already produce part of the attainable risk reduction, reducing the incremental contribution associated with artificial intelligence. Effective policy should therefore coordinate regulatory and technological instruments rather than assume that continually increasing either one will automatically generate proportionally larger benefits.

In conclusion, the results reposition artificial intelligence as a conditional environmental capability rather than an automatic technological solution. Its association with lower pollution-related environmental public health risks is most evident when regions possess the financial resources, infrastructure, institutional capacity, and time required to convert digital development into operational change. The main contribution of this study is therefore not simply to show whether artificial intelligence is beneficial, but to explain when, through which processes, and under what limits that benefit is more likely to emerge. This perspective provides a more realistic basis for incorporating artificial intelligence into environmental public health strategies while recognizing that technology alone cannot immediately reverse persistent pollution structures.

### 6.2 Policy implications

First, environmental policy should prioritize applying artificial intelligence to clearly defined pollution control functions, rather than pursuing digital expansion as an end in itself. The principal fixed-effects results indicate that artificial intelligence is associated with lower pollution-related environmental public health risks, while the dynamic estimates suggest that the timing of its costs and benefits may differ. Public investment should therefore focus on applications that can produce identifiable operational improvements, including continuous emissions monitoring, abnormal pattern detection, pollution forecasting, equipment maintenance, energy scheduling, and production process optimization (20–23, 32, 80, 81). Environmental authorities could establish integrated platforms that connect industrial sensors, environmental monitoring stations, administrative records, and spatial information systems. These platforms should be evaluated through measurable outcomes such as earlier detection of abnormal emissions, shorter response times, reduced energy intensity, and lower releases of specific pollutants. A phased implementation approach is preferable to indiscriminate large-scale deployment. Pilot projects should be assessed through lifecycle energy use, infrastructure costs, pollution outcomes, and operational reliability before being expanded across regions or industries.

Second, artificial intelligence policy should be accompanied by complementary environmental investment. The heterogeneity results show that the negative association of artificial intelligence strengthens as environmental investment rises, and the marginal effects remain significant across representative investment levels. This implies that digital capability alone is insufficient when regions lack monitoring equipment, data infrastructure, cleaner production facilities, skilled personnel, and financial resources for maintenance and implementation. Local governments should therefore coordinate artificial intelligence programs with environmental budgets, green finance, equipment renewal programs, and technical training (29, 31, 40–44, 68). Funding should not be concentrated only on algorithm development or computing capacity. It should also support sensors, pollution treatment equipment, interoperable databases, industrial retrofitting, and personnel capable of converting analytical outputs into environmental action. Regions with limited fiscal capacity may require central transfers, shared technical platforms, or cross-regional service arrangements to prevent artificial intelligence from widening existing differences in environmental management capacity.

Electricity consumption requires a more differentiated policy response. The negative interaction between artificial intelligence and electricity consumption suggests that energy-intensive regions may possess greater technical scope for load forecasting, smart-grid coordination, equipment optimization, and efficiency improvement. However, the conditional effects of artificial intelligence are not statistically significant at representative electricity consumption levels. High electricity demand should therefore not be treated as evidence that artificial intelligence will automatically produce environmental gains. In electricity-intensive regions, intelligent systems should be introduced together with energy efficiency standards, cleaner power sources, demand-side management, and monitoring of data center and computing energy use (32, 45–48, 83). Policy evaluation should distinguish between efficiency improvements produced by intelligent systems and additional electricity demand generated by computing infrastructure. This distinction is particularly important where the regional power mix remains dependent on carbon- and pollution-intensive energy sources.

Third, environmental regulation and artificial intelligence should be coordinated rather than pursued independently. Threshold analysis shows that artificial intelligence remains negatively associated with environmental public health risks in both regulatory regimes, but its coefficient decreases in absolute magnitude after environmental regulation exceeds the estimated threshold. The policy implication is not that governments should continually raise regulatory intensity until a particular numerical threshold is crossed. The threshold value is sample-specific and should not be interpreted as a universal administrative target. Instead, the result indicates that formal regulation and technology-enabled environmental management may partly address the same sources of pollution and operational inefficiency. Under relatively limited regulation, artificial intelligence can add substantial monitoring and optimization capacity. Under stronger regulation, mandatory standards, inspections, and compliance investments may already achieve part of the available improvement, reducing the incremental contribution of artificial intelligence (78, 79). Governments should therefore use adaptive regulatory design, combining risk-based inspections, real-time monitoring, data disclosure, and differentiated compliance requirements. Where intelligent monitoring is reliable, regulatory agencies may use it to target inspections and reduce duplicated administrative costs, while maintaining human oversight and legal accountability.

Fourth, policies directed toward technological expenditure and green innovation should reflect the different empirical stability and timing of these mechanisms. Technological expenditure provides clear evidence of a relatively immediate implementation pathway. Public and private expenditure should therefore be directed toward projects that translate artificial intelligence into operational systems, including pollution sensors, intelligent treatment equipment, environmental software, technical personnel, and industrial energy management platforms (27–32). Funding programs should specify environmental performance indicators and require post-investment evaluation so that technological expenditure is linked to pollution control outcomes.

Green patents require a longer-term and more selective policy approach. The indirect effect through green patents is visible in the pooled analysis but is not statistically stable after persistent provincial and annual differences are controlled. Policy should therefore avoid using the number of green patent applications as the primary measure of successful artificial intelligence-enabled innovation. Greater attention should be given to patent quality, technological relevance, licensing, commercialization, adoption by pollution-intensive firms, and verified environmental performance (33–37, 74, 75). Demonstration projects, green public procurement, technology transfer platforms, and collaboration between patent holders and industrial users may help convert codified inventions into operational pollution control technologies. This approach recognizes that green patents can contribute to environmental improvement, while avoiding the assumption that patent accumulation alone produces an immediate reduction in public health-related environmental pressure.

Finally, the use of artificial intelligence in environmental public health policy should be supported by stronger data integration and institutional safeguards. Because the dependent variable in this study measures pollution-related environmental conditions, environmental authorities and public health agencies should develop secure procedures for linking emissions, ambient environmental quality, population exposure, hospitalization, and disease data. Such integration would allow future policy evaluation to determine whether reductions in measured pollution are followed by improvements in population health. At the same time, intelligent monitoring systems should be governed by clear standards for data quality, privacy, algorithmic transparency, cybersecurity, and human review. Artificial intelligence can improve the speed and precision of environmental decision-making, but responsibility for regulatory enforcement and public health protection should remain with accountable institutions.

### 6.3 Limitations and future research directions

Several limitations define the empirical scope of this study and identify questions that cannot be resolved using the current provincial panel.

First, the environmental public health risk index represents regional pollution-related pressure rather than realized population exposure or observed health outcomes. Carbon dioxide emissions, sulfur dioxide emissions, nitrogen oxide emissions, industrial wastewater discharge, and industrial solid waste provide a multidimensional account of environmental conditions, but they do not reveal which populations were exposed, the duration or intensity of that exposure, or whether it resulted in specific diseases or deaths. Future research should link emissions inventories with satellite observations, meteorological conditions, river basin flows, population distribution, hospitalization records, and cause-specific morbidity and mortality. Such data would permit a sequential examination of whether artificial intelligence development is followed by changes in emissions, population exposure, and ultimately health outcomes. It would also clarify whether equivalent reductions in regional pollution generate different health benefits across densely populated, industrial, rural, and environmentally vulnerable areas.

Second, the annual data do not fully reveal the timing through which artificial intelligence may affect environmental conditions. Digital infrastructure, computing capacity, intelligent equipment, and organizational adjustment can create immediate financial and energy costs, whereas improvements in monitoring, energy efficiency, and pollution control may develop gradually. The difference between the fixed-effects and dynamic estimates indicates that this temporal sequence requires direct investigation. Future studies could use distributed-lag models to estimate effects over several years, local projections to trace pollutant-specific responses over time, and event-study designs around identifiable expansions of computing infrastructure, industrial digitalization programs, or intelligent environmental monitoring systems. These designs would help determine whether the environmental response to artificial intelligence is delayed, cumulative, temporary, or non-linear.

Third, the present mechanism variables do not capture the complete conversion of technological resources into environmental outcomes. Technological expenditure is measured as an aggregate regional input and cannot identify the proportion directed specifically toward intelligent environmental monitoring, cleaner production, pollution treatment, or energy management. Green patent applications similarly represent only the invention stage and do not indicate patent quality, granting, licensing, commercialization, adoption, or verified environmental performance. Future research should combine project-level expenditure, procurement contracts, equipment installation, patent citations, licensing transactions, technology transfer agreements, and firm adoption records. Firm-level designs would be particularly valuable for examining how internal management, environmental accountability, financing constraints, and disclosure practices influence the implementation of artificial intelligence. Research linking artificial intelligence with biodiversity disclosure and financing conditions already demonstrates the relevance of corporate-level governance and accountability for understanding whether digital capability translates into environmental action (84).

Fourth, provincial administrative boundaries do not necessarily correspond to the movement of pollution, electricity, technology, or industrial activity. Air pollutants cross regional borders, wastewater travels through shared river systems, electricity is transmitted through interconnected grids, and technological knowledge diffuses through supply chains and collaborative networks. The current models therefore cannot distinguish local environmental improvement from pollution displacement, imported technological benefits, or cross-regional coordination. Future research should construct spatial and network matrices based on atmospheric transport, river connectivity, electricity transmission, industrial input–output relationships, and technological collaboration. Although developed in a financial context, dynamic return and volatility spillover methods provide a methodological reference for examining how shocks and responses propagate across connected units (85). Environmental applications should adapt these tools to identify whether artificial intelligence investment in one region reduces risks elsewhere, shifts pollution to neighboring regions, or generates benefits through shared infrastructure and technological diffusion.

Finally, the findings are derived from a specific combination of industrial structure, regulatory capacity, energy dependence, environmental investment, and artificial intelligence development. They cannot be transferred directly to economies with substantially different institutional and technological conditions. Future comparative research should therefore move beyond simply enlarging the country sample and instead examine how the relationship changes across institutional regimes. Cross-country panels involving Belt and Road Initiative economies or other developing regions could apply distribution-sensitive methods such as the Method of Moments Quantile Regression to determine whether artificial intelligence has different associations at low and high levels of environmental pressure, as demonstrated in recent international research on heterogeneous environmental effects (5). Comparative designs should also account for institutional quality, green growth capacity, and differences in development conditions, since these factors can alter the distribution and inclusiveness of sustainability outcomes across developing economies (86). Matching countries or regions by energy structure, industrial composition, regulatory enforcement, environmental data capacity, and artificial intelligence maturity would provide a stronger basis for distinguishing context-specific findings from relationships that remain visible across institutional settings.

The next stage should identify the timing of its effects, the pathway from emissions to human exposure, the conversion of technological inputs into implemented solutions, and the geographic and institutional scale at which environmental benefits or costs ultimately emerge.

## Data Availability

The original contributions presented in the study are included in the article/Supplementary material, further inquiries can be directed to the corresponding author.

## Appendix A

### Pooled mediation analysis without provincial and year fixed effects

As a comparative analysis, the indirect effects through technological expenditure and green patents are re-estimated using pooled mediation models without provincial and year fixed effects. These specifications combine persistent cross-sectional differences between provinces with temporal variation occurring within provinces. They therefore do not isolate the within-province mediation processes examined in the main fixed-effects analysis. The pooled models are reported to illustrate how the estimated indirect effects change when time-invariant provincial characteristics and common annual shocks are not removed. As shown in Table A1, the pooled indirect effect through technological expenditure is negative and statistically significant [(IE = −0.450), (*p* = 0.020)]. The bias-corrected 95% confidence interval ranges from (−0.819) to (−0.071) and excludes zero. The pooled indirect effect through green patents is also negative and statistically significant ((IE = -1.024), (*p* < 0.001)), with a bias-corrected 95% confidence interval ranging from (−1.544) to (−0.507). Both indirect effects are therefore statistically significant in the pooled specifications. However, these estimates reflect both between-province differences and within-province changes. Their comparison with the main fixed-effects results suggests that persistent provincial differences contribute substantially to the estimated mediation pathways, particularly the pathway through green patents.

**Table A1:** Pooled Bootstrap mediation estimates without provincial and year fixed effects.

Hypothesis	Mediating path	Indirect effect	(*p*)-value	Bias-corrected 95% CI	Result
H2a	AI → TE → EPHR	−0.450	0.020	[−0.819, −0.071]	Significant in pooled mode
H2b	AI → GP → EPHR	−1.024	<0.001	[−1.544, −0.507]	Significant in pooled model

The indirect effects are estimated using 1,000 pooled Bootstrap resamples. The pooled mediation equations do not include provincial or year fixed effects. The estimates therefore combine persistent between-province differences with temporal variation within provinces. These results are presented only as a comparative analysis and are not used as the primary tests of Hypotheses 2a and 2b. AI denotes artificial intelligence, TE denotes technological expenditure, GP denotes green patents, and EPHR denotes environmental public health risks.
